# PD-1 Expression on *Mycobacterium tuberculosis*-Specific CD4 T Cells Is Associated With Bacterial Load in Human Tuberculosis

**DOI:** 10.3389/fimmu.2018.01995

**Published:** 2018-08-31

**Authors:** Cheryl L. Day, Deborah A. Abrahams, Rubina Bunjun, Lynnett Stone, Marwou de Kock, Gerhard Walzl, Robert J. Wilkinson, Wendy A. Burgers, Willem A. Hanekom

**Affiliations:** ^1^Department of Microbiology and Immunology, Emory University School of Medicine, Atlanta, GA, United States; ^2^Emory Vaccine Center, Emory University, Atlanta, GA, United States; ^3^South African Tuberculosis Vaccine Initiative (SATVI) and School of Child and Adolescent Health, Institute of Infectious Disease and Molecular Medicine, University of Cape Town, Cape Town, South Africa; ^4^Division of Medical Virology, Department of Pathology, Institute for Infectious Disease and Molecular Medicine, University of Cape Town, Cape Town, South Africa; ^5^Division of Molecular Biology and Human Genetics, Faculty of Medicine and Health Sciences, DST/NRF Centre of Excellence for Biomedical Tuberculosis Research, South African Medical Research Council Centre for Tuberculosis Research, Stellenbosch University, Cape Town, South Africa; ^6^Wellcome Centre for Infectious Diseases Research in Africa, Institute for Infectious Disease and Molecular Medicine, University of Cape Town, Cape Town, South Africa; ^7^Tuberculosis Laboratory, Francis Crick Institute, London, United Kingdom; ^8^Department of Medicine, Imperial College London, London, United Kingdom

**Keywords:** CD4 T cell, CD8 T cell, tuberculosis, PD-1, IFN-γ, proliferation

## Abstract

Persistent antigen stimulation in chronic infections has been associated with antigen-specific T cell dysfunction and upregulation of inhibitory receptors, including programmed cell death protein 1 (PD-1). Pulmonary tuberculosis (TB) disease is characterized by high levels of *Mycobacterium tuberculosis* (Mtb), yet the relationship between bacterial load, PD-1 expression, and Mtb-specific T cell function in human TB has not been well-defined. Using peripheral blood samples from adults with LTBI and with pulmonary TB disease, we tested the hypothesis that PD-1 expression is associated with bacterial load and functional capacity of Mtb-specific T cell responses. We found that PD-1 was expressed at significantly higher levels on Th1 cytokine-producing Mtb-specific CD4 T cells from patients with smear-positive TB, compared with smear-negative TB and LTBI, which decreased after completion of anti-TB treatment. By contrast, expression of PD-1 on Mtb-specific CD8 T cells was significantly lower than on Mtb-specific CD4 T cells and did not differ by Mtb infection and disease status. *In vitro* stimulation of PBMC with Mtb antigens demonstrated that PD-1 is induced on proliferating Mtb-specific CD4 T cells and that Th1 cytokine production capacity is preferentially maintained within PD-1^+^ proliferating CD4 T cells, compared with proliferating Mtb-specific CD4 T cells that lack PD-1 expression. Together, these data indicate that expression of PD-1 on Mtb-specific CD4 T cells is indicative of mycobacterial antigen exposure and identifies a population of effector cells with Th1 cytokine production capacity. These studies provide novel insights into the role of the PD-1 pathway in regulating CD4 and CD8 T cell responses in Mtb infection and provide rationale for future studies to evaluate PD-1 expression on antigen-specific CD4 T cells as a potential biomarker for bacterial load and treatment response in human TB.

## Introduction

Infection with *Mycobacterium tuberculosis* (Mtb) is responsible for over 10 million cases of tuberculosis (TB) and approximately 1.7 million deaths each year (1). The number of people who develop active TB disease represents a minority of the estimated 1.7 billion people infected with Mtb who remain asymptomatic and are considered to have latent Mtb infection (LTBI) (2). Infection with Mtb is increasingly recognized to represent a spectrum ranging from eradication of the bacteria, establishment of LTBI, sub-clinical disease, and active TB disease (3). However, the immune correlates of these diverse states of Mtb infection remain poorly understood.

CD4 T cells play an important role in immune containment of Mtb infection. Mtb-infected mice that lack CD4 T cells demonstrate increased susceptibility to TB (4–6), and reactivation of TB is increased following CD4 T cell depletion in macaques with LTBI (7, 8). Moreover, people with LTBI who are co-infected with human immunodeficiency virus (HIV) are at substantially higher risk of developing active TB compared with HIV-uninfected individuals (1, 9–11). In addition to CD4 T cells, CD8 T cells play an important role in containing Mtb infection by release of cytokines and production of cytotoxic molecules such as perforin, granzymes, and granulysin (12–19). Increasing evidence indicates Mtb-specific CD4 and CD8 T cells develop progressive dysfunction in people who develop active TB disease, including decreased IL-2 production (20–23), impaired proliferative capacity (20, 24), and diminished cytolytic activity (25, 26).

Although there is mounting evidence of progressive T cell dysfunction with increasing bacterial load, the mechanisms leading to functional impairment of Mtb-specific T cell responses in people with active TB disease have not been well-defined. One mechanism leading to inhibition of antigen-specific T cell effector function is expression of inhibitory receptors, such as PD-1, CTLA-4, LAG-3, TIM-3, and BTLA, which negatively regulate activated T cells (27). Progressive dysfunction of antigen-specific T cells in tumors and several models of chronic viral infections, including lymphocytic choriomeningitis virus (LCMV), HIV, hepatitis C virus (HCV), and hepatitis B virus (HBV), has been linked to sustained high expression of inhibitory receptors (27, 28). Importantly, antibody-mediated blockade of inhibitory receptor signaling pathways has been shown to enhance antigen-specific T cell function and promote control of infectious pathogens (29), and forms the basis of checkpoint blockade immunotherapy in the treatment of several different cancers (30).

Despite intensive investigation of inhibitory receptor expression by T cells in the settings of tumors and chronic viral infections, expression of inhibitory receptors has been less well-characterized in Mtb infection and TB disease. Pulmonary TB disease in humans is associated with high bacterial loads in the lung, with smear-positive pulmonary TB patients harboring ~10,000 to >10^8^ bacilli per ml of sputum (31, 32). In non-human primates, PD-1 expression is upregulated in tissues of rhesus monkeys with severe TB disease (33). Mtb infection of PD-1^−/−^ mice leads to increased frequencies of Mtb-specific CD4 T cells; however, PD-1^−/−^ mice display enhanced susceptibility to TB disease, characterized by increased bacterial loads, increased inflammatory and necrotic responses in the lungs, and reduced survival (34–36). These studies in PD-1-deficient mice demonstrate a necessary role for PD-1 in limiting excessive IFN-γ production by CD4 T cells, which has been associated with exacerbated disease in murine models of TB (37).

Increasing evidence suggests PD-1 expression is upregulated on innate and adaptive immune cells in the setting of Mtb infection and disease in humans. Expression of PD-1 and its ligands, PD-L1, and PD-L2, has also been reported to be increased on NK cells (38), neutrophils (39), and monocytes (40, 41) from patients with active TB disease. PD-1 has also been reported to be expressed at higher levels on Mtb-specific CD4 T cells in individuals with LTBI, compared to Mtb-uninfected individuals who have been vaccinated with Bacillus Calmette-Guérin (BCG) (42). However, the relationship between bacterial load, PD-1 expression and functional capacity of Mtb-specific T cells in humans remains incompletely understood. To test the hypothesis that PD-1 expression is associated with bacterial load and Mtb-specific T cell functional capacity, we conducted a comprehensive analysis of PD-1 expression on Mtb-specific CD4 and CD8 T cells in clinically well-characterized cohorts of South African adults across a spectrum of Mtb infection and disease.

## Materials and methods

### Study population and sample collection

Participants between the ages of 18–65 years were recruited in the Western Cape province of South Africa; all participants were seronegative for HIV-1 antibodies. Healthy, asymptomatic adults with latent Mtb infection (LTBI) were identified as individuals with no previous history of diagnosis or treatment for TB disease, and with an IFN-γ^+^ T cell response to CFP-10 and/or ESAT-6 antigens by a QuantiFERON®-TB Gold In-Tube assay, or an 8h whole blood intracellular cytokine staining assay (20). Patients with pulmonary TB were diagnosed by standard sputum smear microscopy and/or sputum culture and classified into two groups: sputum smear-negative (smear^−^) and smear-positive (smear^+^). All sputum smear^−^ TB patients had sputum cultures that were positive for Mtb growth. Blood was collected from patients with TB either prior to, or within the first 7 days of starting the standard 6-month course of TB treatment. A subset of TB patients was followed longitudinally on TB treatment, with additional blood samples collected 2 and 6 months after initiating treatment. All TB patients included in the study had been newly diagnosed with TB and reported no previous history of diagnosis or treatment for TB disease. Blood was collected in BD Vacutainer® tubes containing sodium heparin. PBMC were isolated by density centrifugation and frozen in liquid nitrogen until further use.

### Ethics statement

This study was conducted in accordance with the principles expressed in the Declaration of Helsinki. All participants gave written informed consent for the study, which was approved by the institutional review boards at the University of Cape Town, Stellenbosch University, and Emory University; and by the Western Cape Province Department of Health.

### Antigens

The following antigens were used in this study: pools of 15-mer overlapping peptides spanning the full length sequences of CFP-10, ESAT-6, TB10.4, and human cytomegalovirus (HCMV) pp65, pooled by protein (1.25 μg/ml/peptide in whole blood and BAL intracellular cytokine staining assays; 0.1 μg/ml/peptide in 6-day proliferation assays); Mtb purified protein derivative (PPD; 10 μg/ml in whole blood and BAL ICS assays; 1 μg/ml in 6-day proliferation assay; Statens Serum Institut, Denmark); and staphylococcal enterotoxin B (SEB; Sigma-Aldrich) as a positive control (1 μg/ml in whole blood and BAL ICS assays; 0.1 μg/ml in 6-day proliferation assay). The HCMV pp65 peptide pool was obtained through the NIH AIDS Reagent Program, Division of AIDS, NIAID, NIH (43–45).

### Antibodies

The following human monoclonal fluorescently-conjugated antibodies were used in this study: anti-CD3 Pacific Blue (clone UCHT1), anti-CD3 APC-H7 (clone SK7), anti-CD4 APC (clone RPA-T4), anti-CD8 PerCP-Cy5.5 (clone SK-1), anti-IFN-γ Alexa Fluor 700 (clone B27), anti-IL-2 FITC (clone 5344.111), anti-IL-2 APC (clone MQ1-17H12), anti-CCR7 FITC (clone 150503), anti-CD45RA PE-Cy7 (clone HI100), all from BD Biosciences, and anti-CD4 QDot605 (clone S3.5; Life Technologies), anti-TNF-α PE-Cy7 (clone Mab11; eBiosciences), and anti-PD-1 PE (clone EH12.2H7; BioLegend). For PD-1/PD-L1 blockade experiments, LEAF™ purified anti-human PD-L1 Ab (clone 29E.2A3) and LEAF™ purified IgG2b isotype-matched control Ab (clone MPC-11) were obtained from BioLegend.

### Whole blood intracellular cytokine staining (ICS) phenotyping assay

Whole blood ICS assays were performed as previously described (20). Briefly, immediately after collection, whole blood was incubated with antigens (described above); blood incubated with no antigen served as a negative control. Brefeldin A (10 μg/ml; Sigma-Aldrich) was added after 3 h incubation at 37°C, and the incubation continued for an additional 5 h. Blood was diluted in FACS Lysing Solution (BD Biosciences) to lyse red blood cells and fix white blood cells, which were cryopreserved in freezing medium containing 50% RPMI, 40% fetal calf serum (FCS), and 10% dimethyl sulfoxide (DMSO; Sigma-Aldrich), and stored in liquid nitrogen until use.

Cryopreserved, fixed white blood cells were thawed and washed in PBS, followed by a wash with Perm/Wash Buffer (BD Biosciences). For analysis of PD-1 expression and cytokine production profiles, cells were stained for 1 h at 4°C with the following Abs: anti-CD3 Pacific Blue, anti-CD4 QDot605, anti-CD8 PerCP-Cy5.5, anti-IFN-γ Alexa Fluor 700, anti-IL-2 FITC, anti-TNF-α PE-Cy7, and anti-PD-1 PE. For analysis of PD-1 expression and memory T cell phenotypes, cells were stained with anti-CCR7 FITC for 20 min at 37°C, washed with Perm/Wash Buffer, and then stained for 1 h at 4°C with the following Abs: anti-CD3 Pacific Blue, anti-CD4 QDot605, anti-CD8 PerCP-Cy5.5, anti-CD45RA PE-Cy7, anti-PD-1 PE, and anti-IFN-γ Alexa Fluor 700. Cells were then washed in Perm/Wash Buffer and acquired on a BD LSRII flow cytometer.

### Bronchoalveolar lavage (BAL) sample processing

Bronchoscopy was performed on individuals with LTBI according to routine clinical care guidelines and routine bronchoscopy standard operating procedures (46). A flexible standard diagnostic bronchoscopy was conducted under local anesthesia with lidocaine as per British Thoracic Society guidelines. BAL was performed with 160 ml sterile saline installed in aliquots and recovered into a sterile collection bottle. BAL fluid was kept on ice and transported to the laboratory for further processing. BAL fluid was filtered through a 100 μm filter; mononuclear cells were isolated by centrifugation, washed in sterile PBS, and suspended in RPMI containing 10% human male AB serum. BAL mononuclear cells were stimulated under the following conditions: media alone (negative control), a combined CFP-10/ESAT-6 peptide pool, PPD, and SEB. Cells were incubated for 1 h at 37°C, after which Brefeldin A was added (10 μg/ml) and the cells were incubated at 37°C for an additional 5 h. Cells were washed in PBS, stained with LIVE/DEAD Fixable Violet Dead Cell Stain (Vivid; Life Technologies), washed again in PBS, and fixed using FACS Lysing Solution. Cells were washed in PBS and then permeabilized using Perm/Wash Buffer and stained for 1 h at 4°C with the following antibodies: anti-CD3 APC-H7, anti-CD4 APC, anti-CD8 PerCP-Cy5.5, anti-IFN-γ Alexa Fluor 700, anti-IL-2 FITC, anti-TNF-α PE-Cy7, and anti-PD-1 PE. Cells were then washed with Perm/Wash Buffer, suspended in PBS, and acquired on a BD LSRII flow cytometer.

### Proliferation assays and *in vitro* PD-1/PD-L1 blockade

Six-day proliferation assays were performed using freshly isolated PBMC labeled with 0.5 μg/ml CellTrace™ Oregon Green® 488 carboxylic acid diacetate, succinimidyl ester (OG; Life Technologies), as described previously (20). Each stimulation condition (media alone, combined CFP-10/ESAT-6 peptide pool, PPD, and SEB) was set up in duplicate: 10 μg/ml LEAF™ purified anti-human PD-L1 Ab was added to one set of conditions, and 10 μg/ml LEAF™ purified IgG2b isotype-matched control Ab was added to the duplicate set of conditions. Plates were incubated for 6 days in a 37°C incubator with 5% CO_2_. On day 6, 50 μl of cell culture supernatants per well were removed and stored for ELISA analysis (see below). Cells were then suspended in the remaining supernatant and transferred from the 96-well plate to FACS tubes, where they were re-stimulated with antigen for 6 h to determine the capacity of proliferating Mtb-specific CD4 T cells to produce Th1 cytokines following cognate antigen stimulation. Cells were re-stimulated for 6 h on day 6 with the same antigens added on day 0 as follows: 1μg/ml CFP-10/ESAT-6 peptide pool was added to cells stimulated with CFP-10/ESAT-6 peptide pool on day 0; 10 μg/ml PPD was added to cells stimulated with PPD on day 0; 1 μg/ml SEB was added to cells stimulated with SEB on day 0; no antigens were added to cells in the negative control wells containing media alone. Cells were incubated at 37°C for 1 h, then 10 μg/ml Brefeldin A was added, and the cells incubated for an additional 5 h at 37°C. Cells were then washed in PBS, stained with Vivid, fixed with FACS Lysing Solution, permeabilized with Perm/Wash Buffer, and stained at 4°C with the following Abs: anti-CD3 APC-H7, anti-CD4 Qdot605, anti-CD8 PerCP-Cy5.5, anti-PD-1 PE, anti-IFN-γ Alexa Fluor 700, anti-IL-2 APC, and anti-TNF-α PE-Cy7. After 1 h, cells were washed with Perm/Wash Buffer, suspended in PBS, and acquired on a BD LSRII flow cytometer.

### IFN-γ ELISA

Cell culture supernatants from each well were harvested on day 6 of the proliferation assay described above. Supernatants were frozen and stored at −80°C until use in a DuoSet® IFN-γ ELISA (R&D Systems), according to manufacturer's instructions. The limit of detection was 16pg/ml of IFN-γ. Data from antigen-stimulated wells are reported after subtraction of IFN-γ levels in the negative control wells.

### Data analysis

Flow cytometry data were analyzed using FlowJo version 9.6.4 (Tree Star, Inc.). Compensation was calculated using single-stained anti-mouse Ig, κ CompBeads (BD Biosciences). Single cells were gated by plotting forward scatter-area versus forward scatter-height; lymphocytes were gated based on morphological characteristics. Viable cells were defined as Vivid^lo^ cells; proliferating cells were defined as OG^lo^ cells. CD4 T cells were defined as CD3^+^CD4^+^ lymphocytes; CD8 T cells were defined as CD3^+^CD8^+^ lymphocytes.

In whole blood and BAL ICS assays, antigen-specific CD4 and CD8 T cell populations were defined as cells producing cytokines (IFN-γ, TNF-α, and IL-2) after stimulation with antigen. Responses were considered positive if the frequency of cytokine-producing CD4 and CD8 T cells in the antigen-stimulated condition met all of the following criteria: (i) the difference in the absolute number of cytokine^+^ CD4 or CD8 T cells in the antigen-stimulated versus unstimulated condition was ≥20 cytokine^+^ cells, (ii) a minimum frequency of 0.01% of cytokine^+^ CD4 or CD8 T cells, after subtraction of the frequency of cytokine^+^ cells in the negative control, and (iii) a frequency of cytokine^+^ CD4 or CD8 T cells that was greater than the median plus 3 times the median absolute deviation of cytokine^+^ CD4 or CD8 T cells in the negative control samples in the cohort. Phenotypic analysis of PD-1 expression on antigen-specific CD4 and CD8 T cells was restricted to individuals who met the above criteria for a positive response.

In 6-day proliferation assays, proliferative responses were considered positive if the frequency of proliferating (OG^lo^) CD4 or CD8 T cells in the antigen-stimulated wells met both of the following criteria: (i) ≥0.01% of OG^lo^ CD4 or CD8 T cells, after subtraction of the frequency of OG^lo^ cells in the negative control (media alone), and (ii) a frequency of OG^lo^ CD4 or CD8 T cells that was greater than the median plus 3 times the median absolute deviation of OG^lo^ CD4 or CD8 T cells in the negative control samples in the cohort. Analysis of PD-1 expression and cytokine production by proliferating cells was restricted to individuals with positive antigen-specific proliferating CD4 and CD8 T cell responses.

### Statistical analysis

Statistical analysis of data was performed using GraphPad Prism version 7.0b. Differences between three groups were first evaluated using a non-parametric Kruskal-Wallis test, with *p*-values adjusted for multiple comparisons using Dunn's post-test. Differences between two groups were evaluated using a non-parametric Mann-Whitney test. Correlations were evaluated using a non-parametric Spearman rank correlation. Differences in responses within the same individual measured at two different time points on TB treatment were evaluated using the Wilcoxon matched pairs signed rank test. Differences in measurements of T cell function in the presence or absence of anti-PD-L1 blocking Ab were evaluated using the Wilcoxon matched pairs signed rank test. *P*-values < 0.05 were considered significant.

## Results

### Participants

Participants between the ages of 18–65 years were recruited in the Western Cape province of South Africa; all participants were sero-negative for HIV-1 antibodies. Individuals with LTBI were defined as asymptomatic, healthy adults with no previous history of TB diagnosis or treatment, and with IFN-γ^+^ T cell responses to either CFP-10 and/or ESAT-6 peptide pools. Patients with newly diagnosed pulmonary TB disease were categorized into two groups according to sputum smear microscopy results: smear^−^ TB and smear^+^ TB. Both groups of TB patients were older than the LTBI group (Table 1).

**Table 1:** Characteristics of study population.

**Participant group**	***n***	**AFB sputum smear grade**	**Age, years (range)^b^**	**Sex (% male)**
LTBI	48	N/A^a^	25 (18–50)	44
Smear^−^ TB	12	Negative	45 (18–59)^*^	78
Smear^+^ TB	45	3^+^ (*n* = 28); 2^+^ (*n* = 6); 1^+^ (*n* = 11)	38 (18–65)*	61

a *N/A (not applicable)*.

b *Values denote median (range)*.

* *P < 0.05, compared with LTBI*.

### PD-1 expression is increased on subsets of naïve and memory CD4 T cells in patients with smear^+^ TB

**Figure 1:**
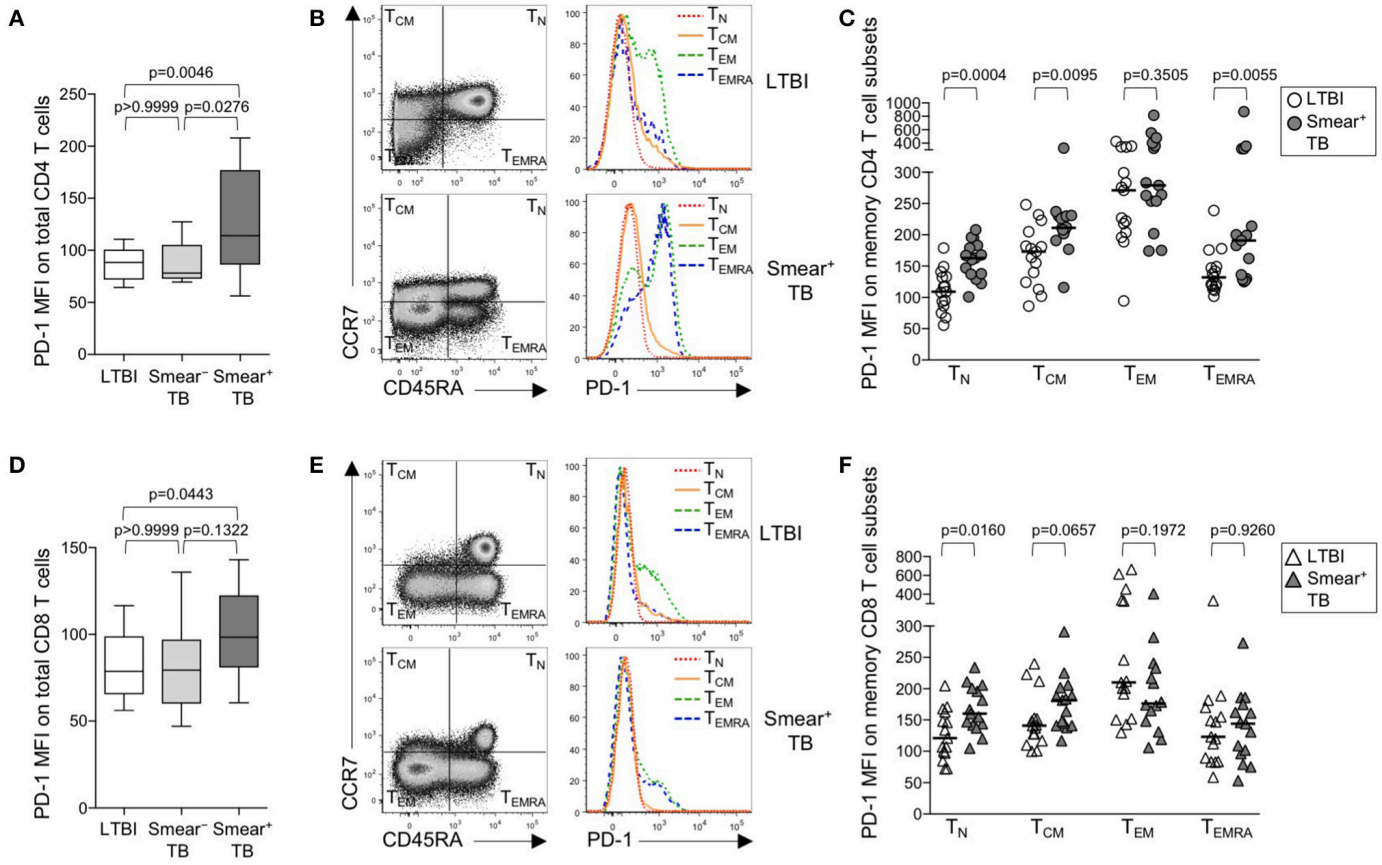
PD-1 is upregulated on naïve and memory CD4 T cell subsets in patients with smear^+^ TB disease. PD-1 expression was measured by flow cytometry using whole blood samples obtained from individuals with LTBI (*n* = 33), and patients with smear^−^ TB (*n* = 12) and with smear^+^ TB (*n* = 29). The MFI of PD-1 expression is shown on the total CD4 T cell population **(A)** and the total CD8 T cell population **(D)**. PD-1 expression on naïve and memory CD4 and CD8 T cell subsets was performed on a subset of individuals with LTBI (*n* = 15) and patients with smear^+^ TB (*n* = 15). Representative flow cytometry data are shown of the naïve and memory T cell profile of CD4 T cells **(B)** and CD8 T cells **(E)** from an individual with LTBI (top row) and a patient with smear^+^ TB (bottom row). The flow cytometry data in **(B)** are gated on viable CD3^+^CD4^+^ lymphocytes; the flow cytometry data shown in **(E)** are gated on viable CD3^+^CD8^+^ lymphocytes. T_N_, T_CM_, T_EM_, and T_EMRA_ subsets are indicated in each quadrant, according to expression of CCR7 and CD45RA. A histogram overlay of PD-1 expression by each T cell subset (T_N_, T_CM_, T_EM_, and T_EMRA_) is shown on the right. Composite data are shown indicating PD-1 MFI on naïve and memory CD4 **(C)** and CD8 T cell subsets **(F)**. Horizontal lines represent the median. Differences between the three groups in **(A, D)** were assessed using a Kruskal-Wallis test; the *p*-values shown have been adjusted for multiple comparisons using Dunn's post-test. Differences between two groups in **(C, F)** were assessed using the Mann-Whitney test. For the box and whiskers plots in **(A, D)**, the horizontal line represents the median, the boxes the interquartile range, and the whiskers the 10th and 90th percentiles.

Upregulation of PD-1 expression by CD4 and CD8 T cells has been associated with T cell dysfunction in the setting of chronic infections, including malaria, HIV, HCV, and HBV (27, 29). We hypothesized that high bacterial loads in people who develop active TB disease would result in upregulation of PD-1 expression on T cells. Using flow cytometry, we measured PD-1 expression on total CD4 and CD8 T cell populations in whole blood from healthy individuals with LTBI, smear^−^ TB patients, and smear^+^ TB patients. PD-1 expression was significantly higher on total CD4 T cells in smear^+^ TB patients, compared with smear^−^ TB patients and individuals with LTBI (Figure 1A). We next evaluated PD-1 expression on naïve and memory CD4 T cell subsets, based on CCR7 and CD45RA expression profiles, in individuals with LTBI and smear^+^ TB patients (Figure 1B). As expected, PD-1 expression was highest on effector memory CD4 T cells (T_EM_; Figure 1C). Compared with individuals with LTBI, PD-1 expression was significantly higher on naïve (T_N_), central memory (T_CM_), and terminally differentiated effector memory cells (T_EMRA_) in patients with smear^+^ TB (Figure 1C).

PD-1 expression by CD8 T cells was higher in smear^+^ TB patients, compared with individuals with LTBI (Figures 1D,E); however, unlike PD-1 expression on CD4 T cells, PD-1 expression was similar between smear^−^ and smear^+^ TB patients. Upregulation of PD-1 on total CD8 T cells in individuals with smear^+^ TB was restricted to the T_N_ CD8 T cells (Figure 1F). Together these data indicate smear^+^ TB disease is associated with upregulation of PD-1 expression on multiple subsets of CD4 T cells, with the greatest increase in expression observed by T_N_ cells.

### PD-1 is upregulated on mtb-specific CD4 T cells in smear^+^ TB patients

**Figure 2:**
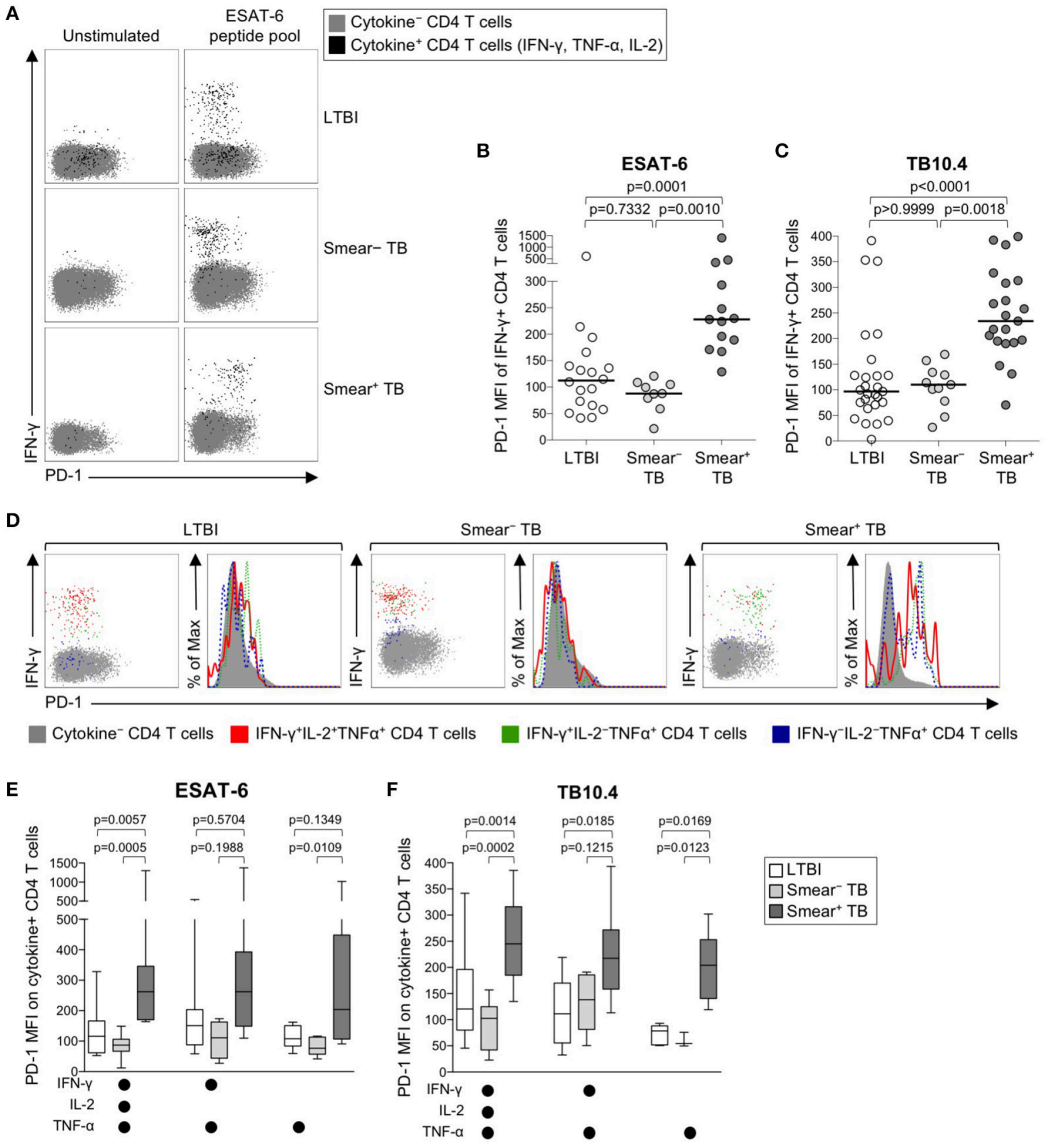
PD-1 expression on Mtb-specific CD4 T cells is increased in patients with smear^+^ TB, compared with smear^−^ TB patients and individuals with LTBI. Whole blood from individuals with LTBI (*n* = 33), and patients with smear^−^ TB (*n* = 12) and smear^+^ TB (*n* = 29), was stimulated for 8 h with an ESAT-6 peptide pool and a TB10.4 peptide pool; PD-1 expression was measured on total CD4 T cells and Ag-specific CD4 T cells producing IFN-γ, TNF-α, and/or IL-2. **(A)** Representative flow cytometry data indicating PD-1 expression by ESAT-6-specific CD4 T cells producing IFN-γ, TNF-α, and/or IL-2. Flow plots are shown gated on viable CD3^+^CD4^+^ lymphocytes; gray cells indicate cytokine^−^ total CD4 T cells and black cells indicate cytokine^+^ (expressing any combination of IFN-γ, TNF-α, and IL-2) CD4 T cells. **(B)** Composite data of PD-1 expression by IFN-γ^+^ CD4 T cells in individuals with a positive response to ESAT-6 (LTBI: *n* = 18; smear^−^ TB: *n* = 9; smear^+^ TB: *n* = 13). **(C)** Composite data of PD-1 expression by IFN-γ^+^ CD4 T cells in individuals with a positive response to TB10.4 (LTBI: *n* = 27; smear^−^ TB: *n* = 11; smear^+^ TB: *n* = 21). **(D)** Representative flow cytometry data indicating PD-1 expression by the indicated subsets of cytokine-producing CD4 T cells following stimulation with ESAT-6. Dot plots and histograms are shown gated on viable CD3^+^CD4^+^ cells. Gray cells indicate cytokine^−^ (IFN-γ^−^ IL-2^−^ TNF-α^−^) CD4 T cells; red indicates IFN-γ^+^ IL-2^+^ TNF-α^+^ cells; green indicates IFN-γ^+^ IL-2^−^ TNF-α^+^ cells; dark blue indicates IFN-γ^−^ IL-2^−^ TNF-α^+^ cells. Dot plots and histograms of PD-1 expression by ESAT-6-specific cytokine^+^ CD4 T cells are shown from the same donor with either LTBI, smear^−^ TB, and smear^+^ TB. **(E,F)** PD-1 expression on subsets of ESAT-6-specific **(E)** or TB10.4-specific **(F)** CD4 T cells producing the indicated combinations of IFN-γ, TNF-α, and IL-2. Only cytokine^+^ subsets present at ≥0.01% of CD4 T cells, after subtraction of background cytokine production, were analyzed for PD-1 expression. Differences across three groups were assessed using a Kruskal-Wallis test; the *p*-values shown have been adjusted for multiple comparisons using Dunn's post-test. Horizontal lines in **(B,C)** represent the median. For box plots in **(E,F)**, the horizontal line represents the median, the box the interquartile range, and the whiskers the 10th and 90th percentiles.

We next sought to determine whether PD-1 was differentially expressed on Mtb-specific CD4 T cells in persons with latent infection and active TB disease. Using a short-term whole blood intracellular cytokine staining (ICS) assay, PD-1 expression was measured on CD4 T cells producing IFN-γ, TNF-α, and/or IL-2 following stimulation with immunodominant Mtb peptide pools (ESAT-6, CFP-10, and TB10.4) and PPD. PD-1 was upregulated on IFN-γ^+^ CD4 T cells in smear^+^ TB patients, compared with smear^−^ TB patients and individuals with LTBI, for each of the Mtb antigens tested (Figures 2A–C, Supplementary Figures 1A, B). No differences in PD-1 expression on Mtb-specific CD4 T cells were found between individuals with LTBI and patients with smear^−^ TB. PD-1 on IFN-γ^+^ CD4 T cells responding to HCMV pp65 (Supplementary Figure 1C) and SEB (Supplementary Figure 1D) was similar between individuals with LTBI and smear^+^ TB patients, thus suggesting upregulation of PD-1 on Mtb-specific CD4 T cells may be driven by exposure to high levels of mycobacterial antigens *in vivo*.

Varying effector functions have been ascribed to antigen-specific T cells producing different combinations of cytokines, with cells producing multiple cytokines simultaneously considered to have greater functional capacity than T cells producing a limited number of cytokines (47–49). To determine whether PD-1 was differentially expressed by functionally distinct subsets of Mtb-specific CD4 T cells, we evaluated PD-1 expression by CD4 T cells producing combinations of IFN-γ, TNF-α, and IL-2 (Figure 2D). Three discrete subsets of ESAT-6- and TB-10.4-specific CD4 T cells were detected, including polyfunctional IFN-γ^+^TNF-α^+^IL-2^+^ cells, IFN-γ^+^TNF-α^+^ double-positive cells, and TNF-α^+^ single-positive cells. Expression of PD-1 was significantly higher on polyfunctional and TNF-α^+^ single-positive subsets of ESAT-6 and TB10.4-specific CD4 T cells in smear^+^ TB patients, compared with individuals with LTBI (Figures 2E,F). These findings were consistent with analysis of PD-1 expression by distinct cytokine^+^ subsets of PPD-specific CD4 T cells, in which PD-1 expression was significantly higher in smear^+^ TB patients, compared with smear^−^ TB and LTBI groups, for each cytokine^+^ subset detected (Supplementary Figure 1E). These data indicate that expression of PD-1 is consistently upregulated on multiple different subsets of Th1 cytokine^+^ Mtb-specific CD4 T cells in patients with newly diagnosed smear^+^ TB disease.

### PD-1 expression is significantly lower on mtb-specific CD8 than CD4 T cells

**Figure 3:**
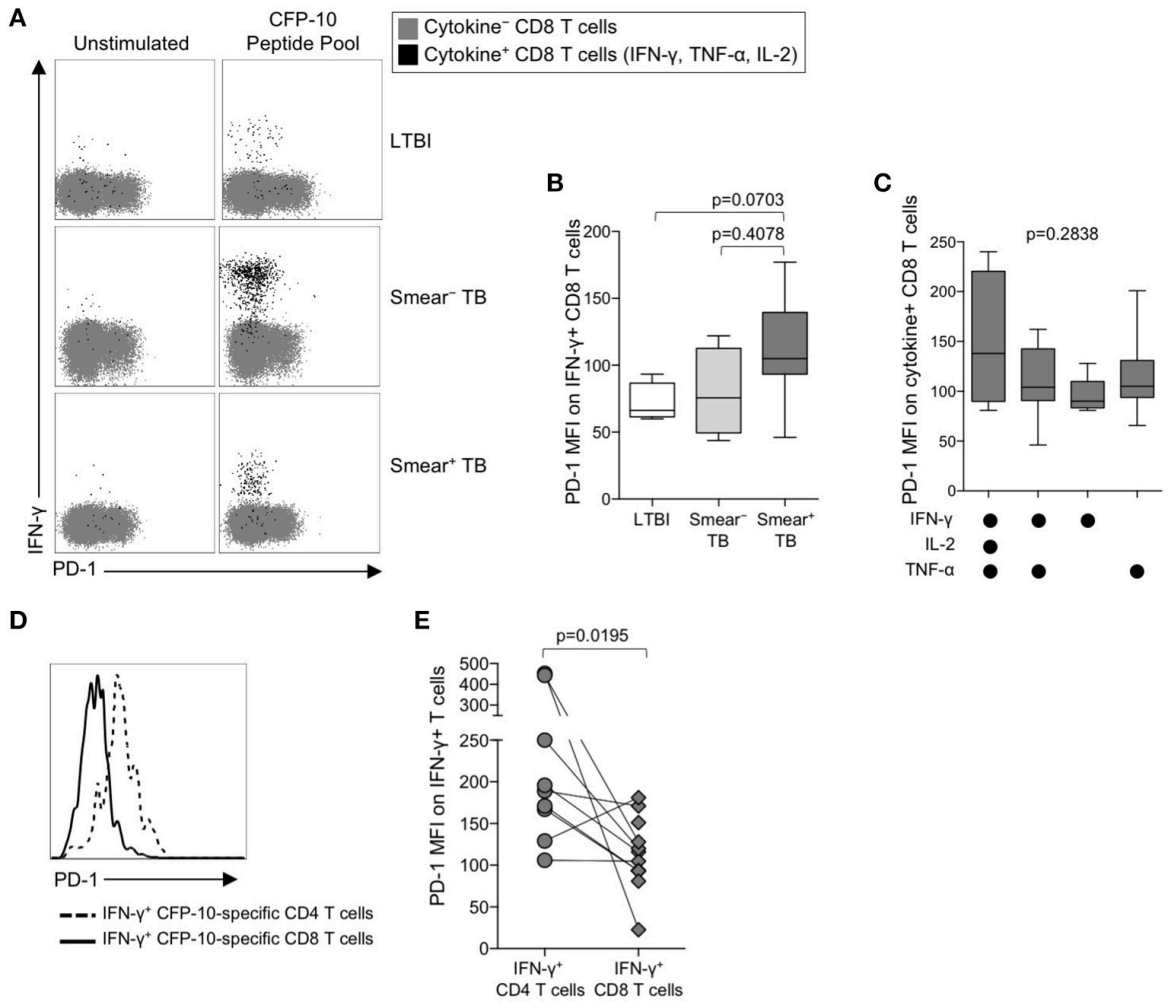
PD-1 is expressed at lower levels by CFP-10/ESAT-6-specific CD8 T cells, compared with CD4 T cells specific for the same antigens. PD-1 expression on CFP-10 and ESAT-6-specific CD8 T cells was performed as described in Figure 2. **(A)** Representative flow cytometry data indicating PD-1 expression by CFP-10-specific CD8 T cells producing IFN-γ, TNF-α, and/or IL-2. Flow plots are shown gated on viable CD3^+^CD8^+^ lymphocytes; gray cells indicate cytokine^−^ CD8 T cells and black cells indicate cytokine-positive CD8 T cells expressing any combination of IFN-γ, TNF-α, and IL-2. **(B)** PD-1 expression by CFP-10/ESAT-6-specific IFN-γ^+^ CD8 T cells (LTBI: *n* = 4; smear^−^ TB: *n* = 4; smear^+^ TB: *n* = 13). Differences were assessed by a Kruskal-Wallis test; the *p*-values shown have been adjusted for multiple comparisons using Dunn's post-test. **(C)** PD-1 expression on subsets of CFP-10/ESAT-6-specific CD8 T cells from smear^+^ TB patients producing the indicated combinations of IFN-γ, TNF-α, and IL-2. Only cytokine^+^ subsets present at ≥ 0.01% of CD8 T cells, after subtraction of background cytokine production, were analyzed for PD-1 expression. No significant differences in PD-1 expression were found between any of the different cytokine-producing subsets (Kruskal-Wallis test). **(D)** Histogram overlay comparison of PD-1 expression on IFN-γ^+^ CD4 and CD8 T cells following stimulation of whole blood from a smear^+^ TB patient with a CFP-10 peptide pool. The dotted line represents PD-1 MFI on IFN-γ^+^ CD4 T cells and the solid line represents PD-1 MFI on IFN-γ^+^ CD8 T cells measured at the same time in the same individual. **(E)** Comparison of PD-1 expression on IFN-γ^+^ CD4 and CD8 T cells specific for the same antigen (either a CFP-10 peptide pool or an ESAT-6 peptide pool) within the same individual (*n* = 9 smear^+^ TB patients). Differences in PD-1 expression between Mtb-specific CD4 and CD8 T cell responses within the same individual were determined by the Wilcoxon matched pairs signed rank test. For box plots in **(B, C)**, the horizontal line represents the median, the box the interquartile range, and the whiskers the 10th and 90th percentiles.

The identification of PD-1 as a marker of T cell dysfunction and exhaustion was initially described in the context of virus-specific CD8 T cells (50), and PD-1 expression on virus-specific CD8 T cells has been correlated with antigen load (51). To determine if PD-1 is upregulated on Mtb-specific CD8 T cells in the setting of chronic Mtb infection, we measured PD-1 expression on cytokine^+^ CFP-10/ESAT-6-specific CD8 T cells in individuals with LTBI, and in smear^−^ and smear^+^ TB patients (Figure 3A). Consistent with previous findings (20, 24, 52, 53), Mtb-specific cytokine^+^ CD8 T cells were detected less frequently in individuals with LTBI than patients with active TB disease. Amongst persons with detectable Mtb-specific, cytokine^+^ CD8 T cell responses, PD-1 expression was not significantly different across the three groups (Figure 3B). Similar to CD4 T cells, there was no evidence of upregulation of PD-1 expression by IFN-γ^+^ CD8 T cells from patients with active TB disease responding to HCMV pp65 (Supplementary Figure 1F) or SEB (Supplementary Figure 1G). Moreover, there was no difference in PD-1 expression by subsets of CFP-10/ESAT-6-specific CD8 T cells producing different combinations of IFN-γ, TNF-α, and IL-2 (Figure 3C).

The above data indicate PD-1 expression is significantly upregulated on Mtb-specific CD4 T cells, but not Mtb-specific CD8 T cells, in smear^+^ TB patients with high bacterial loads. To determine whether PD-1 expression is differentially regulated between Mtb-specific CD4 and CD8 T cells while controlling for bacterial load, we directly compared PD-1 expression on CD4 and CD8 T cell responses specific for the same antigen (either CFP-10 or ESAT-6) within the same individual (Figure 3D). Paired analysis of PD-1 expression on Mtb-specific CD4 and CD8 T cells within the same individual indicated significantly higher expression of PD-1 on Mtb-specific CD4 T cells, compared with Mtb-specific CD8 T cells specific for the same antigen (Figure 3E). Taken together, these data indicate that Mtb-specific CD4 T cells consistently upregulate PD-1 more than Mtb-specific CD8 T cells.

### PD-1 expression by mtb-specific CD4 T cells is similar in BAL and blood of individuals with LTBI

**Figure 4:**
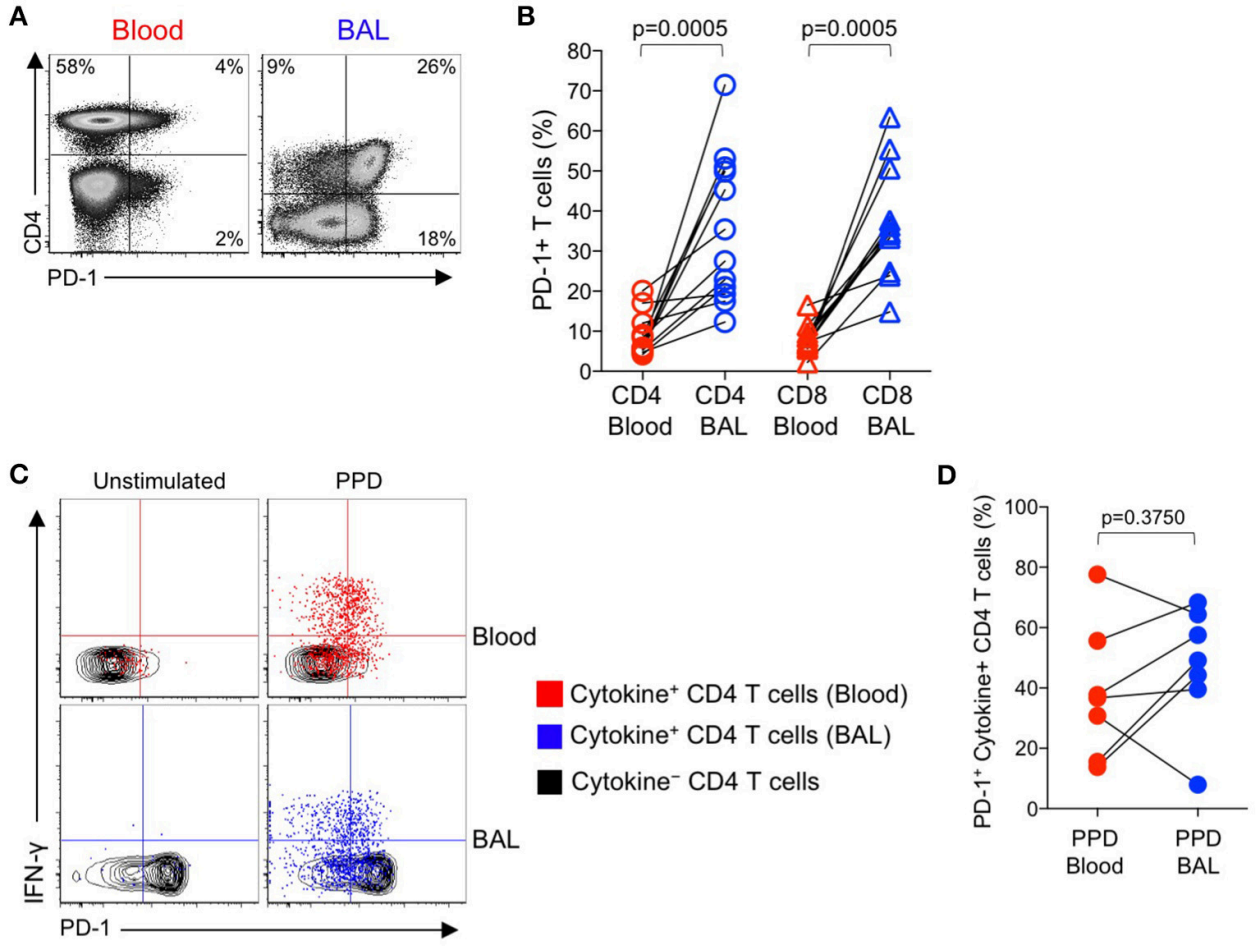
PD-1 expression by PPD-specific CD4 T cells is similar in the lung and peripheral blood of individuals with LTBI. Bronchoalveolar lavage (BAL) was performed on individuals with LTBI (*n* = 12); blood samples collected just prior to the BAL procedure. Whole blood and cells isolated from BAL fluid were stimulated in parallel with PPD for 6 h. PD-1 expression was measured on cytokine^+^ (IFN-γ, TNF-α, and/or IL-2) PPD-specific and total CD4 and CD8 T cells in blood and BAL by flow cytometry. **(A)** Flow cytometry data of PD-1 expression by T cells from paired blood and BAL samples from a single individual with LTBI. Plots are shown gated on viable CD3^+^ T lymphocytes. **(B)** Comparison of PD-1 expression on the total CD4 and CD8 T cell populations in paired blood and BAL samples from individuals with LTBI. **(C)** Flow cytometry data indicating PD-1 expression by cytokine^+^, PPD-specific CD4 T cells from paired blood and BAL samples from two individuals with LTBI. Plots are shown gated on viable CD3^+^CD4^+^ lymphocytes. Cells in gray indicate cytokine^−^ CD4 T cells (for both blood and BAL samples); cells in red indicate PPD-specific CD4 T cells in the blood producing any combination of IFN-γ, TNF-α, and IL-2; cells in blue indicate PPD-specific CD4 T cells in BAL producing any combination of IFN-γ, TNF-α, and IL-2. **(D)** Comparison of PD-1 expression by PPD-specific CD4 T cells producing any combination of IFN-γ, TNF-α, and IL-2 in paired blood and BAL samples from individuals with LTBI (*n* = 7). Differences in **(B, D)** were assessed using the Wilcoxon matched pairs signed rank test.

The above data indicating increased PD-1 expression on Mtb-specific CD4 T cells in smear^+^ TB patients, compared with healthy, asymptomatic individuals with LTBI, were based on analysis of Mtb-specific CD4 T cells circulating in peripheral blood, and thus may not reflect the phenotype of Mtb-specific T cells at the site of infection in the lung. To evaluate PD-1 expression by T cells in the lung and peripheral blood, we performed bronchoalveolar lavage (BAL) on a subset of individuals with LTBI and collected peripheral blood samples from each individual on the same day as the BAL procedure. We first compared PD-1 expression on the total CD4 and CD8 T cell populations in paired blood and BAL samples. For both CD4 and CD8 T cell populations, expression of PD-1 was significantly higher on cells isolated from BAL, compared with peripheral blood from the same individual (Figures 4A,B).

Paired samples of whole blood and BAL were stimulated with PPD and a combined CFP-10/ESAT-6 peptide pool for 6 h, followed by analysis of PD-1 expression on Th1 cytokine^+^ antigen-specific CD4 T cells. The frequencies of CFP-10/ESAT-specific CD4 T cells were too low for phenotyping in BAL; however, cytokine^+^ CD4 T cell responses to PPD were present in BAL at sufficient frequencies for PD-1 phenotypic analysis in 7 out of 12 individuals. Of these individuals with detectable cytokine^+^ CD4 T cell responses in both BAL and blood, PD-1 expression was not significantly different on PPD-specific CD4 T cells in the two compartments (Figures 4C,D). We were unable to detect sufficient frequencies of cytokine^+^ CD8 T cells in BAL samples to evaluate PD-1 expression on Mtb-specific CD8 T cells in BAL and blood (data not shown).

### PD-1 expression on mtb-specific CD4 T cells decreases in smear^+^ TB patients after treatment

**Figure 5:**
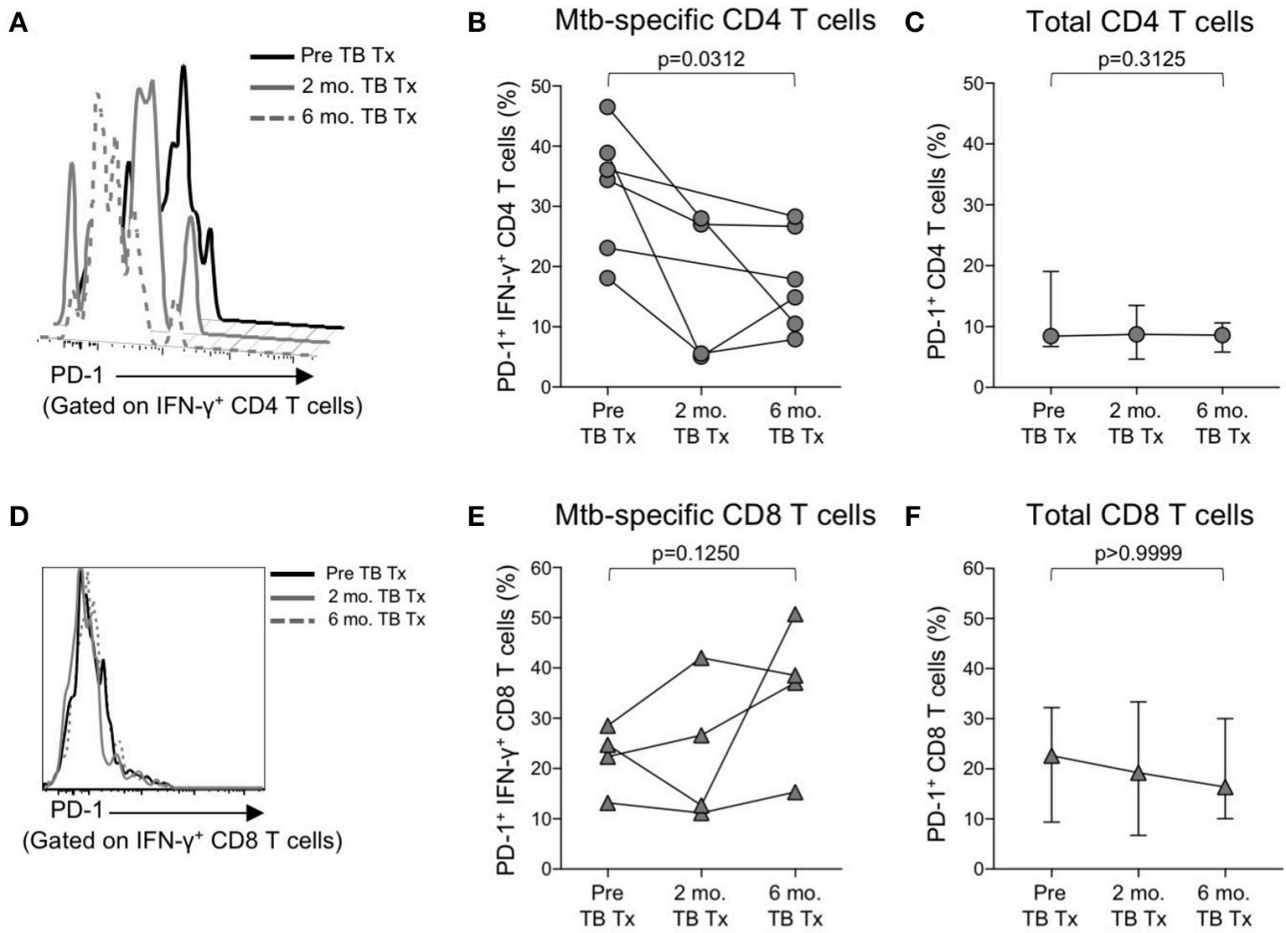
PD-1 expression on Mtb-specific CD4 T cells is associated with bacterial load. Flow cytometry was used to measure PD-1 expression on CFP-10/ESAT-6-specific IFN-γ^+^ CD4 and CD8 T cells in smear^+^ TB patients at 3 time points: prior to initiation of TB treatment (pre-TB Tx), and 2 and 6 months following initiation of treatment. **(A)** Histogram overlay of PD-1 expression on IFN-γ^+^ CD4 T cells following 8 h stimulation of whole blood from a smear^+^ TB patient with a CFP-10 peptide pool at the three time points indicated. PD-1 MFI is shown after gating on viable CD3^+^CD4^+^IFN-γ^+^ lymphocytes. **(B)** Longitudinal analysis of PD-1 expression on CFP-10/ESAT-6-specific IFN-γ^+^ CD4 T cells within the same individuals over time (*n* = 6 smear^+^ TB patients with IFN-γ^+^ CD4 T cell responses detectable at more than one time point). **(C)** Median and interquartile range of PD-1 expression on total CD4 T cells from the same smear^+^ TB patients shown in **(B)**. **(D)** Histogram overlay of PD-1 expression by IFN-γ^+^ CD8 T cells following 8 h stimulation of whole blood from a smear^+^ TB patient with a CFP-10 peptide pool at the three time points indicated. PD-1 MFI is shown after gating on viable CD3^+^CD8^+^IFN-γ^+^ lymphocytes. **(E)** Longitudinal analysis of PD-1 expression on CFP-10/ESAT-6-specific IFN-γ^+^ CD8 T cells within the same individual over time (*n* = 4 smear^+^ TB patients with IFN-γ^+^ CD8 T cell responses detectable at more than one time point). **(F)** Median and interquartile range of PD-1 expression on total CD8 T cells from the same smear^+^ TB patients shown in **(E)**. For **(B,C,E,F)**, differences in PD-1 expression prior to (pre-TB Tx) and at the end of TB treatment (6 mo. TB Tx) were determined using the Wilcoxon matched pairs signed rank test.

We observed increased levels of PD-1 expression by Mtb-specific CD4 T cells in patients with smear^+^ TB disease, but not in patients with smear^−^ TB disease, thus suggesting PD-1 expression on CD4 T cells may reflect bacterial load and thus relate to the level of mycobacterial antigen exposure. To directly assess whether PD-1 expression on Mtb-specific CD4 T cells is associated with bacterial load, we measured PD-1 expression longitudinally on CFP-10 and ESAT-6-specific CD4 T cells on a subset of smear^+^ TB patients during TB treatment. By the end of the 6-month TB treatment period, PD-1 expression on IFN-γ^+^ CD4 T cells had significantly decreased, compared with pre-treatment levels (Figures 5A,B). By contrast, no significant differences were observed in PD-1 expression by CFP-10 and ESAT-6-specific, IFN-γ^+^ CD8 T cells in smear^+^ TB patients after completion of treatment (Figures 5D,E). PD-1 expression on the total CD4 and CD8 T cell populations did not significantly change following completion of anti-TB treatment (Figures 5C,F). Taken together, these data provide further evidence that high bacterial loads in patients with smear^+^ TB disease drive increased expression of PD-1 on Mtb-specific CD4 T cells, and that PD-1 expression decreases on Mtb-specific CD4 T cells following successful treatment of pulmonary TB.

### Blockade of PD-1/PD-L1 signaling *in vitro* augments mtb-specific IFN-γ production

**Figure 6:**
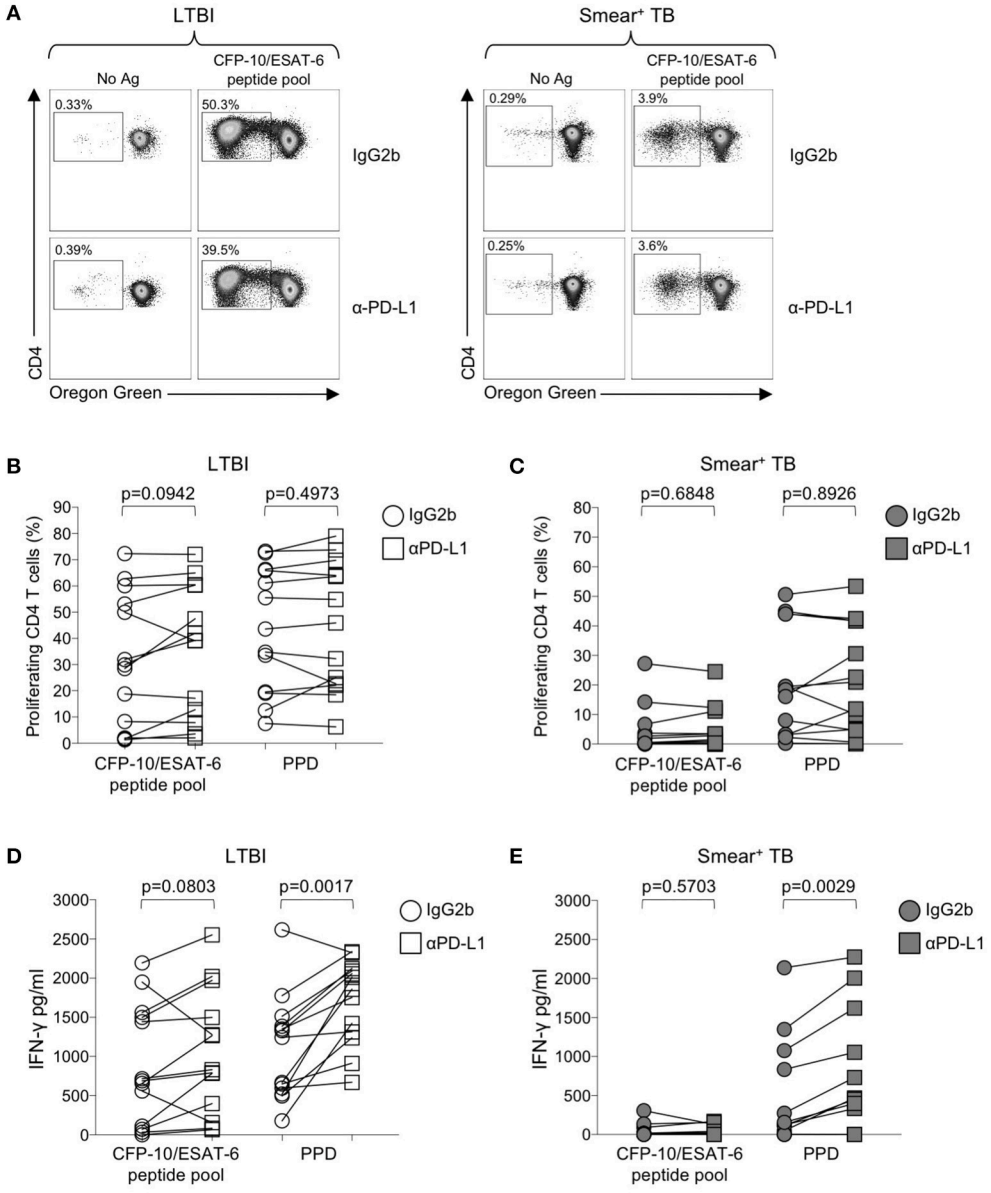
Blockade of the PD-1/PD-L1 signaling pathway can augment Mtb-specific IFN-γ secretion, but not CD4 T cell proliferative capacity. Proliferation assays were performed using freshly isolated PBMC labeled with Oregon Green (OG) and incubated for 6 days under the following conditions: media alone (negative control), CFP-10/ESAT-6 peptide pool, and SEB (positive control). Proliferation assays were performed in the presence of either anti-PD-L1 blocking Ab, or an IgG2b isotype-matched control Ab (*n* = 13 LTBI; *n* = 13 smear^+^ TB). **(A)** Flow cytometry data indicating the frequency of proliferating CD4 T cells following stimulation with CFP-10/ESAT-6 peptide pool, in the presence of anti-PD-L1 Ab or IgG2b isotype control Ab. Dot plots are shown gated on viable CD3^+^CD4^+^ lymphocytes from an individual with LTBI (left) and a patient with smear^+^ TB (right). **(B,C)** Comparison of the frequency of proliferating (OG^lo^) CD4 T cells stimulated with CFP-10/ESAT-6 peptide pool, in the presence of anti-PD-L1 Ab or IgG2b isotype control Ab. **(D,E)** Levels of IFN-γ in cell culture supernatants of PBMC stimulated with CFP-10/ESAT-6 peptide pool for 6 days in the presence of anti-PD-L1 Ab or IgG2b isotype control Ab. Levels of IFN-γ were quantified by ELISA; data are shown after subtraction of background IFN-γ production in the negative control (media alone) conditions. Differences in proliferative capacity and IFN-γ secretion in the presence and absence of anti-PD-L1 Ab were determined using the Wilcoxon matched pairs signed rank test.

Numerous studies in chronic viral infections have indicated virus-specific CD4 and CD8 T cell function, including cytokine production and proliferation, can be augmented or restored following blockade of the PD-1 signaling pathway, either in animal studies *in vivo* (50, 54, 55), or in human studies *in vitro* (51, 56, 57). We have previously reported that Mtb-specific T cell proliferative capacity is impaired in patients with active TB disease (20, 24). To determine whether blockade of the PD-1/PD-L1 signaling pathway *in vitro* could restore or augment the proliferative capacity of Mtb-specific CD4 T cells in patients with smear^+^ TB, PBMC were labeled with the cytosolic dye Oregon Green (OG) and cultured for 6 days with Mtb antigens in the presence of an anti-PD-L1 blocking Ab or an isotype-matched control Ab (Figure 6A). Blockade of the PD-1/PD-L1 pathway enhanced CD4 T cell viability in PBMC cultured for 6 days with media alone, although this difference was abrogated when cells were stimulated with Mtb antigens (Supplementary Figure 2). No differences in the proliferative capacity of Mtb-specific CD4 T were found in the presence of anti-PD-L1 blocking Ab, compared with an isotype control Ab, for either the LTBI (Figure 6B) or smear^+^ TB groups (Figure 6C). Together these data indicate that *in vitro* blockade of the PD-1/PD-L1 signaling pathway may promote CD4 T cell survival but is not sufficient to overcome the dysfunction in proliferative capacity of Mtb-specific CD4 T cells in patients with smear^+^ TB disease.

We next evaluated the capacity of PD-1/PD-L1 blockade to augment cytokine production by Mtb-specific T cells. Using an ELISA for measurement of IFN-γ in cell culture supernatants collected on day 6 of the proliferation assay, we found that PD-1/PD-L1 blockade enhanced PPD-induced IFN-γ production in both LTBI and smear^+^ TB groups (Figures 6D,E). However, PD-1/PD-L1 blockade did not significantly augment IFN-γ production to CFP-10/ESAT-6 peptide pool stimulation in either group. These data indicate the capacity of PD-1/PD-L1 blockade to enhance Mtb-specific IFN-γ production is not dependent on Mtb infection status.

### Expression of PD-1 is induced on proliferating mtb-specific T cells following *in vitro* culture with mtb antigens

**Figure 7:**
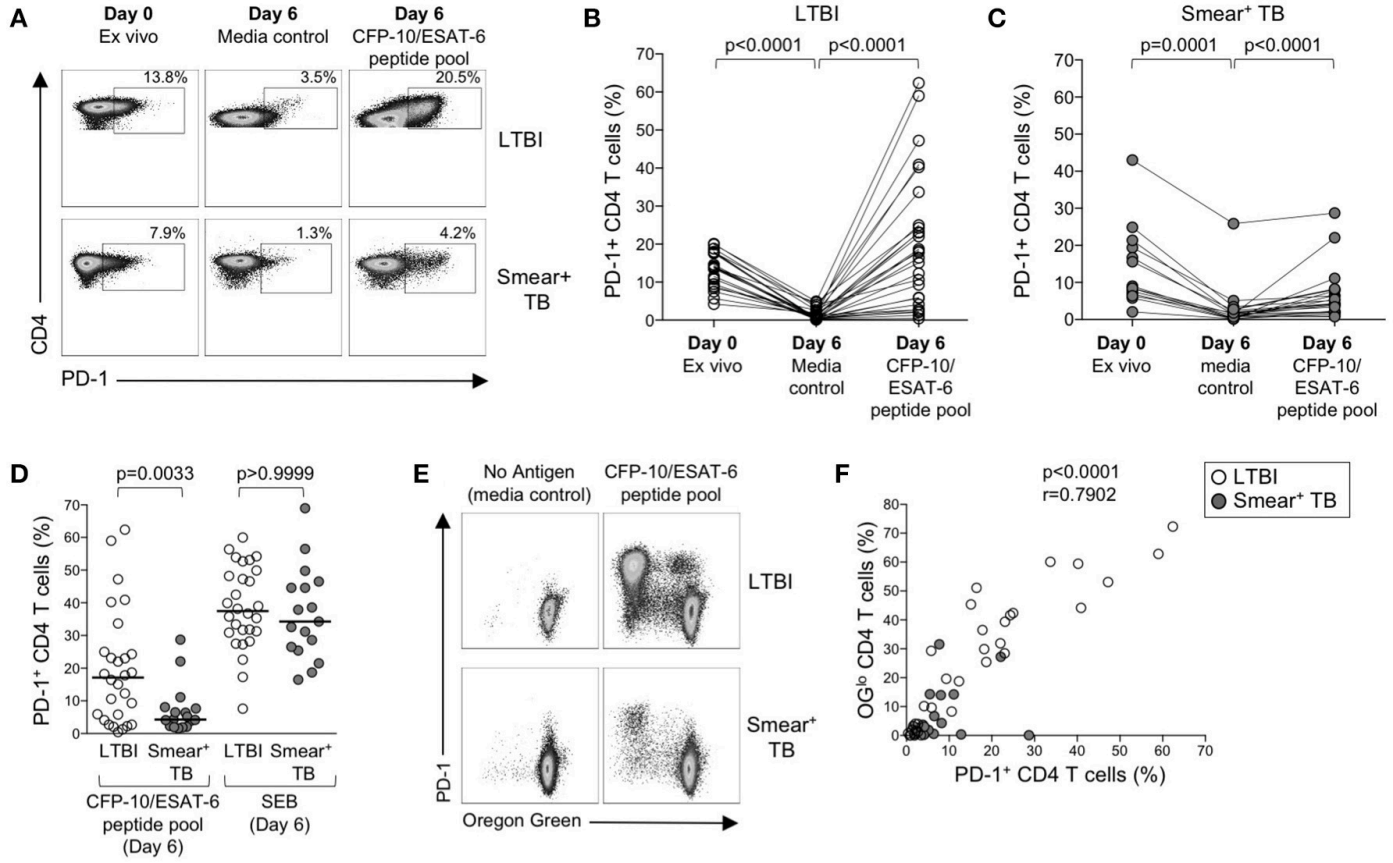
PD-1 expression is induced on CFP-10/ESAT-6-specific CD4 T cells proliferating *in vitro*. Proliferation assays performed as described in Figure 6. PD-1 expression was measured by flow cytometry on total CD4 T cells and CFP-10/ESAT-6-specific proliferating (OG^lo^) CD4 T cells on day 6 of culture. **(A)** Flow cytometry data indicating PD-1 expression on total CD4 T cells in whole blood directly *ex vivo* (Day 0, *ex vivo*), and PD-1 expression on total CD4 T cells after incubation for 6 days in the absence of exogenous Ag stimulation (Day 6, media control), and after stimulation with CFP-10/ESAT-6 peptide pool for 6 days (Day 6, CFP-10/ESAT-6 peptide pool). Flow plots are shown gated on viable CD3^+^CD4^+^ lymphocytes. The percentages of PD-1^+^ CD4 T cells are shown in each plot. Each of the three flow plots shown are from a single individual (top row: LTBI; bottom row: smear^+^ TB). **(B,C)** Composite data indicating the percentage of PD-1^+^ CD4 T cells in each indicated condition for individuals with LTBI [**(B)**, *n* = 28] and patients with smear^+^ TB [**(C)**, *n* = 23]. Differences between PD-1 expression by CD4 T cells in each condition were determined by the Wilcoxon matched pairs rank sum test. **(D)** Comparison of the frequency of PD-1^+^ CD4 T cells in individuals with LTBI and smear^+^ TB patients following stimulation with CFP-10/ESAT-6 peptide pool and SEB. Horizontal lines represent the median. Differences between the two groups were determined using a Mann-Whitney test. **(E)** Flow cytometry data indicating expression of PD-1 by proliferating (OG^lo^) CFP-10/ESAT-6-specific CD4 T cells. Flow plots are shown gated on viable CD3^+^CD4^+^ lymphocytes; data are shown from an individual with LTBI (top row) and a patient with smear^+^ TB (bottom row). **(F)** Correlation between the frequency of proliferating (OG^lo^) CD4 T cells and the frequency of PD-1^+^ CD4 T cells in individuals with LTBI (open circles) and smear^+^ TB patients (gray circles), following stimulation of PBMC with CFP-10/ESAT-6 peptide pools. Significance was determined using the Spearman rank correlation.

The above data suggest that PD-1/PD-L1 blockade can increase Mtb-specific IFN-γ production in both groups of individuals with LTBI and active TB, but does not augment Mtb-specific T cell proliferative capacity in smear^+^ TB patients. To further evaluate the relationship between PD-1 expression and Mtb-specific T cell proliferative capacity, we measured PD-1 on CD4 T cells in whole blood directly *ex vivo*, and on PBMC cultured for 6 days with media alone, CFP-10/ESAT-6 peptide pool, and SEB (Figure 7A). In the absence of exogenous antigen stimulation, PD-1 expression decreased significantly on CD4 T cells in PBMC cultured in media alone, compared with PD-1 expression on CD4 T cells circulating in peripheral blood *ex vivo* (Figure 7B). In both LTBI and smear^+^ TB groups, incubation of PBMC for 6 days with CFP-10/ESAT-6 peptides induced expression of PD-1 at significantly higher levels than PBMC cultured in media alone (Figures 7B,C). However, stimulation with CFP-10/ESAT-6 peptides induced PD-1 expression on a significantly greater percentage of CD4 T cells in individuals with LTBI, compared with smear^+^ TB patients (Figure 7D). The differential induction of PD-1 expression by CD4 T cells in LTBI and smear^+^ TB was particular to Mtb antigen stimulation, as indicated by the similar levels of PD-1 expression induced by CD4 T cells in the two groups following mitogenic stimulation with SEB (Figure 7D).

We next determined whether PD-1 was differentially expressed by proliferating (OG^lo^) and non-proliferating CD4 T cells (Figure 7E). The majority of CD4 T cells that proliferated to CFP-10/ESAT-6 peptides also expressed PD-1, both in individuals with LTBI and in smear^+^ TB patients (Figure 7E). Moreover, a significant and strong positive correlation was found between the frequencies of proliferating CD4 T cells and PD-1^+^ CD4 T cells in CFP-10/ESAT-6-stimulated PBMC cultures (Figure 7F). Taken together, these data suggest that exposure to Mtb antigens *in vitro* induces expression of PD-1 on proliferating Mtb-specific CD4 T cells. By contrast with Mtb-induced PD-1 expression on CD4 T cells, PD-1 expression was relatively low on CD8 T cells cultured for 6 days with CFP-10/ESAT-6 peptide pool and did not differ between individuals with LTBI and smear^+^ TB, with the majority of proliferating CFP-10/ESAT-6-specific CD8 T cells lacking PD-1 expression (data not shown). These data provide further evidence of differential regulation of PD-1 expression on Mtb-specific CD4 and CD8 T cells.

### Mtb-specific CD4 T cells that proliferate also upregulate PD-1 and maintain robust Th1 antigen-specific cytokine production capacity

**Figure 8:**
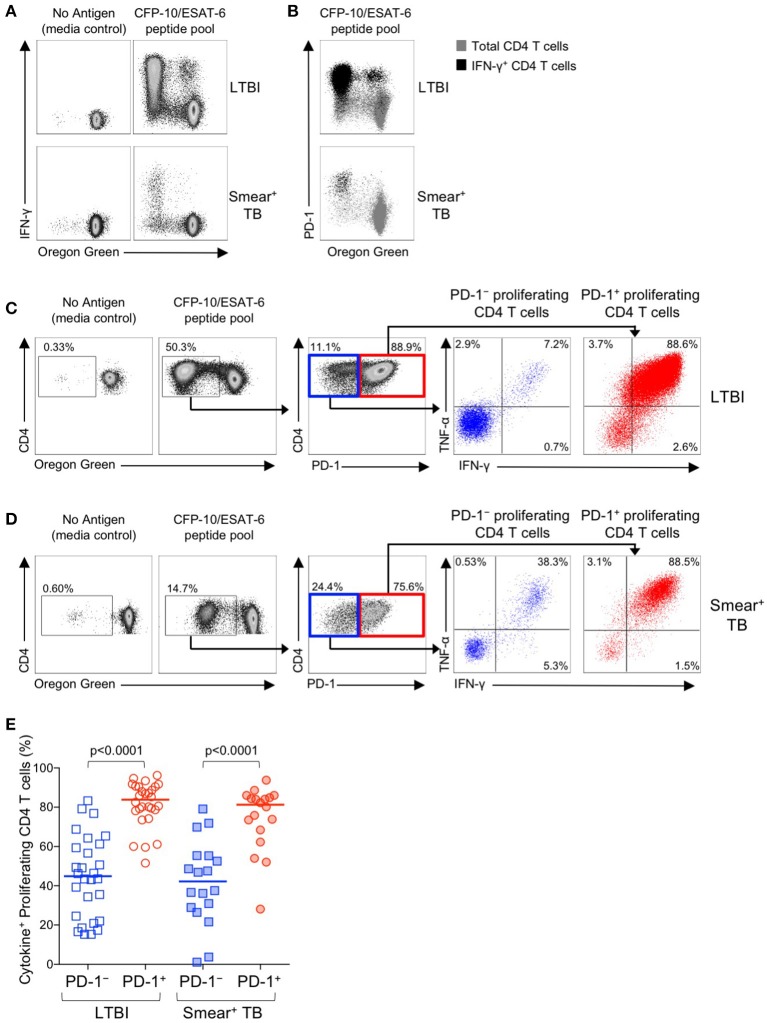
PD-1^+^ proliferating Mtb-specific CD4 T cells exhibit greater antigen-specific cytokine production capacity than PD-1^−^ proliferating cells. PBMC proliferation assays were performed as described in Figure 6. On day 6 of the assay, cells were re-stimulated with a CFP-10/ESAT-6 peptide pool for 6 h, and then analyzed by flow cytometry for expression of PD-1 and intracellular IFN-γ and TNF-α. **(A)** Flow cytometry data indicating IFN-γ production capacity by proliferating CD4 T cells following incubation of OG-labeled PBMC for 6 days in medium alone, or with a CFP-10/ESAT-6 peptide pool. Cells were re-stimulated with the same peptide pool for 6 h on day 6 to measure cytokine production capacity. Plots are shown gated on viable CD3^+^CD4^+^ lymphocytes; data are shown from an individual with LTBI (top row) and a patient with smear^+^ TB (bottom row). **(B)** Flow cytometry data indicating co-expression of PD-1 and IFN-γ by OG^lo^ CD4 T cells following stimulation with a CFP-10/ESAT-6 peptide pool. Plots are shown gated on viable CD3^+^CD4^+^ lymphocytes; data are shown from an individual with LTBI (top row) and a patient with smear^+^ TB (bottom row). Gray cells indicate total CD4 T cells; black cells indicate IFN-γ^+^ CD4 T cells. **(C,D)** Flow cytometry data indicating the gating strategy for analysis of cytokine production capacity by PD-1^−^ and PD-1^+^ proliferating CFP-10/ESAT-6-specific CD4 T cells. Cells were first gated on viable CD3^+^CD4^+^ lymphocytes, then on proliferating (OG^lo^) CD4 T cells, which were then gated on PD-1^−^ and PD-1^+^ cells, and further analyzed for intracellular IFN-γ and TNF-α production. Data shown in **(C)** are from an individual with LTBI; data shown in **(D)** are from a patient with smear^+^ TB. **(E)** Proportion of CFP-10/ESAT-6-specific PD-1^−^ and PD-1^+^ proliferating CD4 T cells that produce IFN-γ and/or TNF-α following re-stimulation with CFP-10/ESAT-6 peptide pools for the final 6 h of the 6-day proliferation assay. Only individuals with CFP-10/ESAT-6-specific CD4 T cell proliferation above background were included in this analysis (*n* = 28 LTBI; *n* = 18 smear^+^ TB). Horizontal lines represent the median. Differences between cytokine production capacity of PD-1^−^ and PD-1^+^ proliferating CD4 T cells was determined using the Wilcoxon matched pairs signed rank test.

We next examined the functional capacity of Mtb-specific CD4 T cells that upregulated expression of PD-1 during the 6-day *in vitro* culture with Mtb peptide pools. To determine whether induction of PD-1 expression *in vitro* was associated with evidence of Mtb-specific CD4 T cell dysfunction, OG-labeled PBMC were re-stimulated with CFP-10/ESAT-6 peptides for the final 6 h of the 6-day culture. Cells were analyzed by flow cytometry to measure proliferation, PD-1 expression, and intracellular IFN-γ and TNF-α expression. Proliferating Mtb-specific CD4 T cells retained the capacity to produce Th1 cytokines upon antigen re-stimulation on day 6 of culture (Figure 8A). Interestingly, the majority of IFN-γ^+^ proliferating CD4 T cells were also PD-1^+^ (Figure 8B).

To determine whether PD-1^+^ versus PD-1^−^ proliferating CD4 T cells exhibit differential cytokine production capacity, cells were first gated on Mtb-specific proliferating cells (OG^lo^CD4^+^), then gated on PD-1^+^ and PD-1^−^ populations and evaluated for intracellular IFN-γ and TNF-α production (Figures 8C,D). In both groups of individuals with LTBI and smear^+^ TB, Mtb-specific proliferating CD4 T cells that expressed PD-1 maintained significantly greater cytokine production capacity than PD-1^−^ CD4 T cells (Figure 8E). Together these data indicate that *in vitro* culture with Mtb antigens induces PD-1 expression by the majority of proliferating Mtb-specific CD4 T cells, and that PD-1^+^ proliferating CD4 T cells retain robust capacity to produce Th1 effector cytokines.

## Discussion

Sustained high expression of PD-1 has been associated with T cell dysfunction in chronic viral infections and tumors (29, 30). Active pulmonary TB disease is characterized by high bacterial loads in the lung, yet the relationship between bacterial load, PD-1 expression, and Mtb-specific T cell function in human TB has not been well-defined. Using well-characterized cohorts of adults in South Africa with LTBI and with newly-diagnosed smear^−^ and smear^+^ pulmonary TB disease, we have made the following key findings: (i) PD-1 expression is upregulated on total CD4 T cells and multiple different Th1 cytokine-producing subsets of Mtb-specific CD4 T cells in patients with smear^+^ TB, compared with smear^−^ TB patients and asymptomatic individuals with LTBI; (ii) PD-1 expression on Mtb-specific CD4 T cells decreases in patients with smear^+^ TB following completion of anti-TB treatment; (iii) *in vitro* blockade of the PD-1/PD-L1 signaling pathway enhances Mtb-specific IFN-γ production, but is insufficient to restore robust Mtb-specific CD4 T cell proliferative capacity in patients with smear^+^ TB; and (iv) *in vitro* culture of PBMC with Mtb antigens induces PD-1 expression on Mtb-specific CD4 T cells that proliferate and have robust Th1 cytokine production capacity.

Our observations that PD-1 is upregulated on Mtb-specific CD4 T cells in smear^+^ TB patients are consistent with previous studies of human chronic viral infections, which indicate that PD-1 is upregulated on virus-specific T cells and correlates with viral load (51, 58–62). Our data are also consistent with a previous study in mice reporting that PD-1 is expressed at high levels on ESAT-6-specific CD4 T cells following Mtb infection (34). Interestingly, PD-1 was significantly upregulated on Mtb-specific CD4 T cells from patients with smear^+^ but not smear^−^ TB disease, suggesting that bacterial load in particular drives increased PD-1, and that the inflammatory milieu of pulmonary TB disease alone is insufficient to significantly increase PD-1 expression. The smear^+^ TB patients in our cohort were older than in our LTBI cohort (Table 1) and we found a weak positive correlation between age and PD-1 expression on total CD4 T cells (*p* = 0.04, *r* = 0.24; data not shown); however, we found no significant correlation between age and PD-1 expression on Mtb-specific CD4 or CD8 T cells (data not shown). Further evidence that PD-1 on Mtb-specific CD4 T cells is driven by bacterial load is provided by our longitudinal studies indicating that expression of PD-1 on Mtb-specific CD4 T cells from patients with smear^+^ TB disease is significantly reduced following successful completion of anti-TB treatment. PD-1 expression on the total CD4 T cell population in this subset of TB patients did not change significantly over time with anti-TB treatment, thus indicating that upregulation of PD-1 on Mtb-specific CD4 T cells from smear^+^ TB patients is driven by cognate antigen recognition. These results are consistent with a previous study reporting that concomitant antiretroviral therapy and TB treatment in HIV-infected TB patients resulted in a reduction of PD-1 expression on PPD-specific CD4 T cells (63). Additional longitudinal studies of larger cohorts of HIV-uninfected patients with active TB disease are required to closely monitor PD-1 expression on Mtb-specific CD4 T cells to evaluate the utility of PD-1 as a biomarker for treatment response, and as a potential predictor of relapse of reinfection.

By contrast with human viral infections in which upregulation of PD-1 has been widely reported on virus-specific CD8 T cells, we found that expression of PD-1 on Mtb-specific CD8 T cells is similar across individuals with LTBI, smear^−^ TB and smear^+^ TB, consistent with a previous report that expression of PD-1 on Mtb-specific CD8 T cells is not significantly different between individuals with LTBI and active TB disease (53). By directly comparing PD-1 expression on Mtb-specific CD4 and CD8 T cells within the same individual, we were able to demonstrate significantly higher PD-1 expression on CD4 T cells, compared with CD8 T cells specific for the same Mtb antigen within the same individual. The lower level of PD-1 expression on Mtb-specific CD8 T cells, compared with CD4 T cells, may reflect lower levels of Mtb antigen presentation by MHC class I molecules, relative to MHC class II molecules in Mtb-infected cells, and potentially differential recognition of Mtb-infected macrophages between antigen-specific CD4 and CD8 T cells, as indicated by recent studies in mice (64). Taken together, these provide further evidence that CD4 and CD8 T cell responses are differentially generated and regulated in Mtb infection.

While our data support the conclusion that PD-1 expression on Mtb-specific CD4 T cells is driven by bacterial load, it is important to note that these data were generated by analysis of Mtb-specific CD4 T cells circulating in peripheral blood. Given that Mtb infects alveolar macrophages in the lung, we hypothesized that higher Mtb antigen exposure in the lung may promote higher expression of PD-1 on Mtb-specific CD4 T cells in the lung than in peripheral blood. Consistent with previous reports of PD-1 expression on T cells in paired BAL and blood samples (65, 66), PD-1 was expressed at higher levels on total CD4 and CD8 T cells in BAL from individuals with LTBI, compared with peripheral blood. Importantly, we demonstrate that PD-1 expression on Mtb-specific CD4 T cells in BAL is similar to that observed on Mtb-specific CD4 T cells circulating in peripheral blood. Unfortunately, due to infection control precautions, we were unable to conduct BAL on patients with smear^+^ TB within the first 7 days of anti-TB treatment, our defined cutoff period of sample collection from patients with active TB disease. Nevertheless, our findings of similar levels of PD-1 expression on Mtb-specific CD4 T cells in BAL and blood provide an important novel insight into PD-1 expression in human Mtb infection and further validate our findings in peripheral blood that PD-1 expression is differentially expressed on Mtb-specific CD4 T cells in individuals with LTBI and patients with smear^+^ TB disease.

The discovery that immunoregulatory molecules such as PD-1 and CTLA-4 are amenable to therapeutic manipulation by antibodies that block these signaling pathways has revolutionized cancer immunotherapy (67–71), and is a potential avenue for novel approaches in the treatment of infectious diseases (29). The potential for PD-1 blockade to improve disease outcomes was the rationale for previous studies of Mtb infection in PD-1^−/−^ mice. Surprisingly, mice that lack PD-1 are exceptionally sensitive to TB, with increased bacterial loads, exacerbated pathology and decreased survival, compared with wild-type mice (34–36). These important studies in mice provide compelling evidence that inhibition of T cells by PD-1 is necessary to prevent excess IFN-γ production and detrimental immune-mediated pathology. Consistent with the *in vivo* studies of Mtb-infected PD-1^−/−^ mice, our *in vitro* studies indicated that PPD-specific IFN-γ production is increased following blockade of the PD-1/PD-L1 signaling pathway. Increased PPD-induced IFN-γ production was observed in both LTBI and smear^+^ TB groups, suggesting that the capacity of PD-1/PD-L1 blockade to augment Mtb-specific IFN-γ production is not unique to Mtb infection status and is not directly correlated with *ex vivo* expression of PD-1, which was significantly lower on PPD-specific CD4 T cells in individuals with LTBI, compared with smear^+^ TB. Alternatively, the capacity of PD-1/PD-L1 blockade to modulate Mtb-specific functional capacity could be more directly related to surface expression of PD-L1, which we did not evaluate in our cohort. Additionally, since we measured IFN-γ alone as a readout of Mtb-specific cytokine production, we cannot preclude the possibility that other cytokines or T cell effector functions, such as cytotoxicity, are differentially modulated in LTBI and smear^+^ TB patients following PD-1/PD-L1 blockade.

Our experimental approach to *in vitro* blockade of the PD-1/PD-L1 pathway utilized anti-PD-L1 antibodies, as we and others have previously used in *in vitro* studies of PD-1 signaling in chronic human viral infections (51, 58–60, 62, 72). A small number of previous studies have conducted *in vitro* blockade of PD-1/PD-L1 in PBMC from TB patients, which have utilized either anti-PD-1 antibodies or a combination of anti-PD-L1 and anti-PD-L2 antibodies (40, 41, 73). While one study reported that blockade of PD-1/PD-L1 signaling during a short-term 6 h stimulation with Mtb did not significantly enhance Mtb-specific IFN-γ production (40), our results are consistent with two previous studies of stimulation of PBMC with Mtb antigens in the presence of PD-1/PD-L1 blockade for ≥3 days (41, 73), which demonstrate Mtb-specific IFN-γ production can be enhanced by blocking PD-1 signaling pathway. Taken together, these *in vitro* studies of *in vitro* PD-1/PD-L1 blockade using PBMC from TB patients are consistent with increased IFN-γ production observed in Mtb-infected PD-1^−/−^ mice (34, 35). Given the exacerbated pathology and increased mortality in Mtb-infected PD-1-deficient mice, it is plausible that enhancing Mtb-specific T cell IFN-γ production by PD-1 blockade in Mtb-infected individuals could ultimately have detrimental effects, as suggested by recent case reports of reactivation of TB in cancer patients treated with anti-PD-1 immunotherapy (74–76).

Despite an increase in Mtb-specific IFN-γ production in both LTBI and smear^+^ TB groups, we did not observe any significant enhancement of Mtb-specific CD4 T cell proliferative capacity with PD-1/PD-L1 blockade, a function that we and other have previously reported to be impaired in patients with smear^+^ TB (20, 24, 77). By conducting in-depth analysis of PD-1 expression profiles in our proliferation assays, we determined that expression of PD-1 is significantly downregulated, compared with *ex vivo* expression, on resting CD4 T cells cultured for 6 days in the absence of exogenous antigen stimulation. By contrast, PD-1 expression is induced at high levels on Mtb-specific CD4 T cells that have proliferated in response to 6-day Mtb antigen stimulation. These data provide additional support for the notion that PD-1 expression is indicative of recently activated Mtb-specific effector CD4 T cells, and are consistent with transient upregulation of PD-1 on antigen-specific T cells described in acute infections (78). Our *in vitro* findings of Mtb antigen-induced PD-1 expression further corroborate our *ex vivo* findings that PD-1 is significantly upregulated on Mtb-specific CD4 T cells in the setting of high bacterial load in smear^+^ TB, indicating recent Mtb antigen exposure *in vivo*, compared with LTBI individuals who have either cleared Mtb or maintain bacterial loads below the level of detection by sputum culture.

Regarding the functional capacity of CD4 T cells that express PD-1, our *in vitro* studies clearly demonstrated that cytokine production capacity is preferentially contained within the subset of proliferating Mtb-specific CD4 T cells that express PD-1, compared with proliferating cells that lack PD-1 expression. These data are also consistent with our *ex vivo* observations that PD-1^+^ Mtb-specific CD4 T cells possess polyfunctional cytokine production capacity (Figure 2). These findings are in line with a recent report that a subset of PD-1^hi^ CD4 T cells have greater cytokine production capacity than PD-1^low^ CD4 T cells in healthy individuals (79), and a report that PD-1^+^ CD4 T cells in HIV-infected children preferentially maintain robust Th1 and Th17 cytokine production, compared with PD-1^−^ CD4 T cells (80). Our findings thus support a growing body of evidence that PD-1 expression alone does not necessarily identify antigen-specific CD4 T cells with impaired functional capacity.

It is important to note that Mtb-specific CD4 T cells from patients with smear^+^ TB disease had higher PD-1 expression *ex vivo* and reduced proliferative capacity *in vitro*, compared with individuals with LTBI, yet Mtb-specific CD4 T cells from smear^+^ TB that had proliferated also expressed PD-1 (Figure 8). Our data indicate that the majority of proliferating Mtb-specific CD4 T cells express PD-1 on day 6 of our proliferation assays, in both LTBI and smear^+^ TB patients; however, it remains unclear whether the proliferating Mtb-specific CD4 T cell population originates from cells that expressed PD-1 *ex vivo*, or whether Mtb-specific CD4 T cells with proliferative capacity induced PD-1 expression only transiently during the 6-day antigen stimulation period. Our data from individuals with LTBI, who express low levels of PD-1 on Mtb-specific CD4 T cells *ex vivo*, strongly indicate that PD-1 is induced on proliferating cells during 6-day antigen stimulation. Future studies in which PBMC are first sorted into PD-1^+^ and PD-1^−^ populations prior to incubation with Mtb antigens in proliferation assays would be necessary to definitely determine whether proliferative capacity is preferentially maintained within PD-1^+^ and PD-1^−^ CD4 T cell populations.

In summary, we have conducted a detailed study of PD-1 expression profiles and effector functions on Mtb-specific CD4 and CD8 T cells in individuals with LTBI and active TB disease. Overall, our data indicate that expression of PD-1 on Mtb-specific CD4 T cells, but not CD8 T cells, defines a population of effector cells with Th1 cytokine production capacity that have recently encountered cognate antigen. These studies provide novel insights into the role of the PD-1 pathway in regulating T cell responses in Mtb infection and provide a platform for future studies to determine the utility of PD-1 expression on antigen-specific CD4 T cells as a biomarker for bacterial load and treatment response in human TB.

## Author contributions

CD, WB, and WH contributed conception and design of the study. DA and RB performed experimental work. CD, WB, and WH contributed to execution and oversight of experimental work. CD contributed to data interpretation, statistical analyses, and drafted the manuscript. LS, MdK, GW, and RW contributed reagents, materials, and/or analysis tools. All authors approved the final manuscript.

### Conflict of interest statement

The authors declare that the research was conducted in the absence of any commercial or financial relationships that could be construed as a potential conflict of interest.
