# Supplementary material for: PD-1 Expression on Mycobacterium tuberculosis-Specific CD4 T Cells Is Associated With Bacterial Load in Human Tuberculosis
Source: Front Immunol. 2018 Aug 31;9:1995. doi: 10.3389/fimmu.2018.01995 (PMC6127207; doi:10.3389/fimmu.2018.01995)
Supplement: Supplementary file 1 [file Data_Sheet_1.PDF]

Supplementary Material

PD-1 expression on *Mycobacterium tuberculosis*-specific CD4 T cells is associated with bacterial load in human tuberculosis

Cheryl L. Day*, Deborah A. Abrahams, Rubina Bunjun, Lynnett Stone, Marwou de Kock, Gerhard Walzl, Robert J. Wilkinson, Wendy A. Burgers, and Willem A. Hanekom

***Correspondence**: Cheryl L. Day: cday@emory.edu

1. **Supplementary Figures**

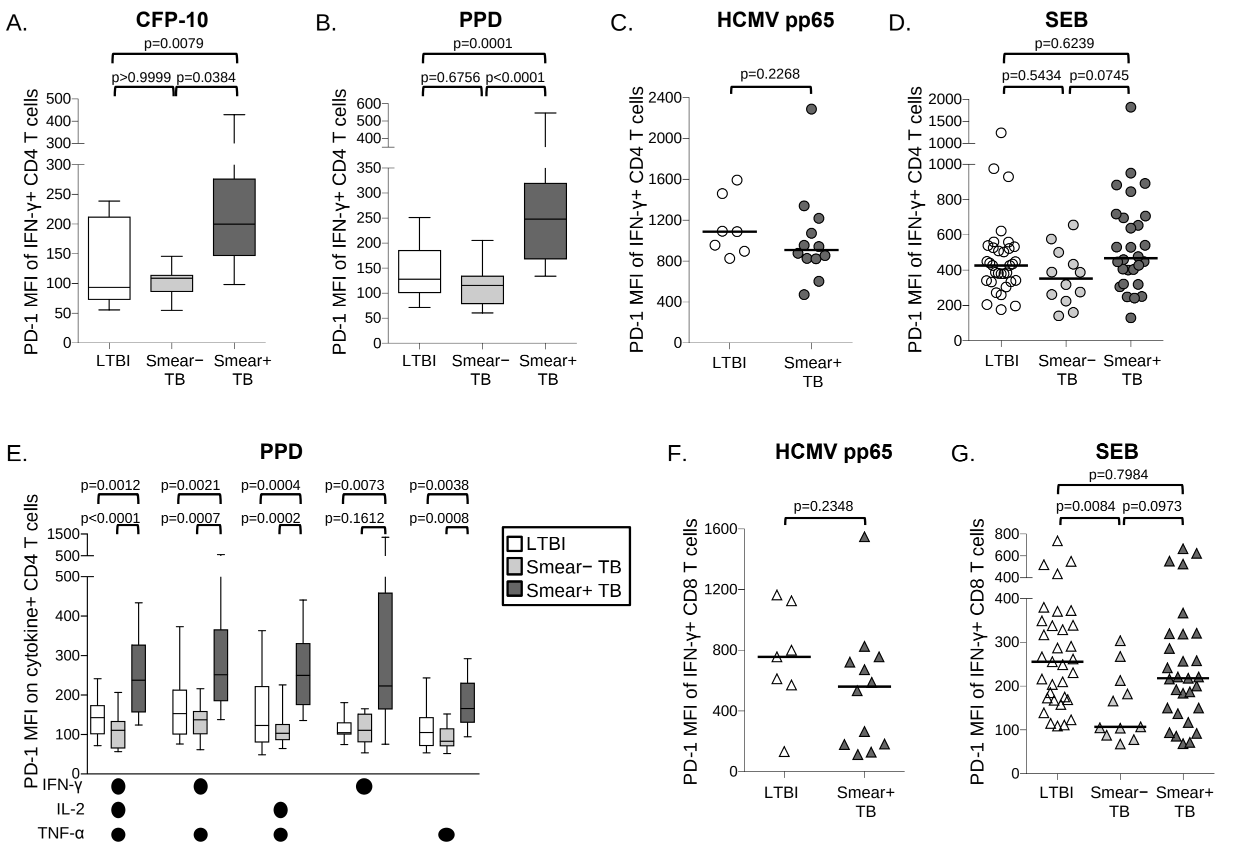

Supplementary Figure 1: PD-1 is upregulated on Mtb-specific CD4 T cells in patients with smear^{+} TB disease. Whole blood from individuals with LTBI and smear^{-} and smear^{+} TB disease was stimulated with antigens as described in Figure 2. The MFI of PD-1 was measured by flow cytometry on cytokine^{+} Ag-specific CD4 and CD8 T cells. (**A**) PD-1 expression on CFP-10-specific CD4 T cells in individuals with LTBI (n=20), smear^{-} TB (n=7) and smear^{+} TB (n=15). (**B**) PD-1 expression on PPD-specific CD4 T cells in individuals with LTBI (n=33), smear^{-} TB (n=12) and smear^{+} TB (n=29). (**C**) PD-1 expression on PPD-specific CD4 T cells expressing the indicated combinations of IFN-γ, IL-2, and TNF-α. (**D**) PD-1 expression on HCMV pp65-specific CD4 T cells in individuals with LTBI (n=7) and smear^{+} TB (n=12). (**E**) PD-1 expression on SEB-stimulated CD4 T cells in individuals with LTBI (n=33), smear^{-} TB (n=12), and smear^{+} TB (n=28). (**F**) PD-1 expression on HCMV pp65-specific CD8 T cells in individuals with LTBI (n=7) and smear^{+} TB (n=12). (**G**) PD-1 expression on SEB-stimulated CD8 T cells in individuals with LTBI (n=33), smear^{-} TB (n=11), and smear^{+} TB (n=29). For panels A, B, and E, the horizontal line represents the median, the box the interquartile range, and the whiskers the 10th and 90th percentiles. Differences between two groups in panels C and F were assessed using the Mann-Whitney test. Differences across three groups were assessed using a Kruskal-Wallis test; the p values shown have been adjusted for multiple comparisons using Dunn's post-test.

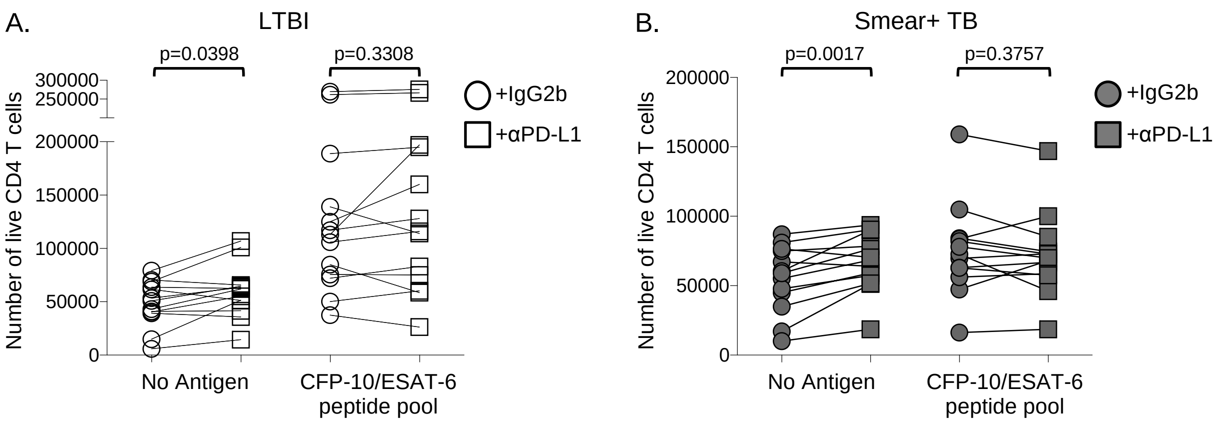

Supplementary Figure 2: Blockade of the PD-1/PD-L1 pathway enhances survival of total CD4 T cells in individuals with LTBI and patients with smear$^+$ TB. Proliferation assays were performed using freshly isolated PBMCs as described in Figure 6. Proliferation assays were performed in the presence of either anti-PD-L1 blocking Ab, or an IgG2b isotype-matched control Ab (n=13 individuals with LTBI; n=13 smear$^+$ TB patients). Equal numbers of PBMCs were added to each condition at day 0 and all cells in each condition were acquired by flow cytometry on day 6 of the assay. The total numbers of viable (Vivid^{lo}) CD3$^+$CD4$^+$ T cells acquired in the unstimulated (PMBCs in media alone) and CFP-10/ESAT-6 peptide pool-stimulated PBMCs are shown from individuals with LTBI (**A**) and patients with smear$^+$ TB disease (**B**). Differences between the number of live CD4 T cells in the IgG2b isotype control and anti-PD-L1 blocking Ab conditions were determined using the Wilcoxon matched pairs rank test.
